# Mortality among Pesticide Applicators Exposed to Chlorpyrifos in the Agricultural Health Study

**DOI:** 10.1289/ehp.9662

**Published:** 2007-01-11

**Authors:** Won Jin Lee, Michael C.R. Alavanja, Jane A. Hoppin, Jennifer A. Rusiecki, Freya Kamel, Aaron Blair, Dale P. Sandler

**Affiliations:** 1 Department of Preventive Medicine, College of Medicine, Korea University, Seoul, Korea; 2 Division of Cancer Epidemiology and Genetics, National Cancer Institute, National Institutes of Health, Department of Health and Human Services, Rockville, Maryland, USA; 3 Epidemiology Branch, National Institute of Environmental Health Sciences, National Institutes of Health, Department of Health and Human Services, Research Triangle Park, North Carolina, USA; 4 Department of Preventive Medicine and Biometrics, Uniformed Services University of the Health Sciences, Bethesda, Maryland, USA

**Keywords:** chlorpyrifos, farmers, injuries, insecticides, mortality, suicide

## Abstract

**Background:**

Chlorpyrifos is one of the most widely used organophosphate insecticides in the United States. Although the toxicity of chlorpyrifos has been extensively studied in animals, the epidemiologic data are limited.

**Objective:**

To evaluate whether agricultural chlorpyrifos exposure was associated with mortality, we examined deaths among pesticide applicators in the Agricultural Health Study, a prospective study of licensed pesticide applicators in Iowa and North Carolina.

**Methods:**

A total of 55,071 pesticide applicators were included in this analysis. Detailed pesticide exposure data and other information were obtained from self-administered questionnaires completed at the time of enrollment (1993–1997). Lifetime chlorpyrifos use was divided into tertiles. Poisson regression analysis was used to evaluate the exposure–response relationships between chlorpyrifos use and causes of death after adjustment for potential confounders.

**Results:**

A total of 1,851 deaths (588 among chlorpyrifos users) were observed during the study period, 1993–2001. The relative risk (RR) of death from all causes combined among applicators exposed to chlorpyrifos was slightly lower than that for nonexposed applicators (RR = 0.90; 95% confidence interval, 0.81–1.01). For most causes of death analyzed, there was no evidence of an exposure–response relationship. However, the relative risks for mortality from suicide and non-motor-vehicle accidents were increased 2-fold in the highest category of chlorpyrifos exposure days.

**Conclusions:**

Our findings of a possible association between chlorpyrifos use and external causes of death were based on small numbers. However, the findings may reflect a link between chlorpyrifos and depression or other neurobehavioral symptoms that deserves further evaluation.

Chlorpyrifos [*O,O*-diethyl-*O*-(3,5,6-trichloro-2-pyridyl)-phosphorothioate] is one of the most widely used organophosphate insecticides in the United States (Kiely et al. 2004). Although the toxicity of chlorpyrifos has been studied extensively in animals, epidemiologic data are limited. A study of Dow Chemical Company employees occupationally exposed to chlorpyrifos from 1977 to 1985 (Brenner et al. 1989) and up to 1994 (Burns et al. 1998) did not identify any potential risks due to chlorpyrifos. However, case–control studies have shown increased risk of non-Hodgkin lymphoma (Waddell et al. 2001) and glioma (Lee et al. 2005) among male farmers exposed to chlorpyrifos. Recently, we reported increased lung cancer incidence associated with exposure to chlorpyrifos in the Agricultural Health Study (Lee et al. 2004).

Although farmers and pesticide applicators have a lower mortality rate for all causes combined than the general population (Blair et al. 2005; Fleming et al. 2003; Stiernstrom et al. 2001), excess mortality has been reported for accidents (Fleming et al. 2003), nonmalignant respiratory diseases (Schenker et al. 1998), and some cancers (Blair and Zahm 1995). Few studies have focused on specific chemicals. Previous studies were limited by small sample sizes and inability to adequately control for potential confounding.

The Agricultural Health Study is a prospective study (Alavanja et al. 1996) designed to examine a wide variety of occupational exposures and lifestyle factors among farmers and commercial pesticide applicators and the risk of cancer and other chronic diseases. To evaluate whether agricultural chlorpyrifos exposure was associated with mortality, we examined deaths among pesticide applicators in this cohort.

## Materials and Methods

### Study population and follow-up

Details of the study design and population have been described previously (Alavanja et al. 1996; Lee et al. 2004). In brief, a total of 57,111 pesticide applicators enrolled in the study in 1993–1997 by completing a questionnaire when they sought a restricted-use pesticide license from the State Cooperative Extension Services or Departments of Agriculture in Iowa and North Carolina. This represented approximately 84% of eligible applicators in both states. Deaths among cohort members were identified through the National Death Index and state death registries for Iowa and North Carolina from time of enrollment through 31 December 2001. Underlying causes of death were coded according to the *International Classification of Diseases* (ICD) *9th Revision* (1993–1999; World Health Organization 1977) and *10th Revision* (2000–2001; World Health Organization 1992). All codes were converted to the *10th Revision* (ICD-10) for statistical analysis (Timely Data Resources 2006). The average follow-up was 6.4 years. All participants provided implied informed consent by returning self-completed questionnaires and verbal informed consent for telephone interviews at follow-up. Institutional review boards of the National Institutes of Health, Battelle Centers for Public Health Research and Evaluation (the field station in North Carolina), the University of Iowa (the field station in Iowa), and Westat (coordinating center for the study) approved the protocol.

### Exposure assessment

Through a self-administered enrollment questionnaire we collected comprehensive exposure data on 22 pesticides and ever/never use information on 28 additional pesticides, as well as information on use of personal protective equipment, pesticide application methods, pesticide mixing, equipment repair, smoking history, alcohol consumption, medical conditions, other lifestyle factors, and basic demographic data. The questionnaire may be found online at http://www.aghealth.org (Agricultural Health Study 2006). Using published pesticide exposure data, we developed the following algorithm to estimate the intensity of exposure to individual pesticides from questionnaire responses: intensity level = (mixing status + application method + equipment repair status) × personal protective equipment use, where the various levels of the four elements of the intensity score were weighted to reflect their importance in determining exposure (Dosemeci et al. 2002). Mixing status was a three-level variable based on never mixing, mixing < 50% of the time, and mixing at least 50% of the time (values of 0, 3, and 9, respectively). Application method was a six-level variable, based on never applying, use of aerial aircraft or distribution of tablets, application in furrow, use of boom on tractor, use of backpack, and use of hand spray (values of 0, 1, 2, 3, 8, and 9, respectively). Equipment repair status was a two-level variable, based on not repairing or repairing pesticide application equipment (0 and 2, respectively). Personal protective equipment use was an eight-level variable based on the types and percentage of time that personal protective equipment was used while applying pesticides. Because skin exposure is believed to be the main route of pesticide absorption, the intensity level algorithm used to estimate pesticide exposure in this study emphasizes dermal absorption.

We constructed two lifetime chlorpyrifos exposure variables for this analysis, each categorized into tertiles, based on the distribution of exposure among all deceased chlorpyrifos-exposed applicators. Lifetime exposure-days was based on the product of the mid-points of the questionnaire categories of number of years an applicator personally applied or mixed chlorpyrifos and number of days in an average year an applicator personally mixed or applied chlorpyrifos (i.e., years of use × days per year, resulting in the following tertiles: ≤ 20.0, 20.1–56.0, ≥ 56.1). Intensity-weighted exposure-days were based on multiplying the lifetime exposure-days by the intensity level (i.e., years of use × days per year × intensity level, resulting in the following tertiles: ≤ 78.8, 78.9–318.1, ≥ 318.2).

### Statistical analysis

Participants who did not provide information on chlorpyrifos exposure (*n* = 1,901) or who were not resident in Iowa or North Carolina at the time of enrollment (*n* = 339) were excluded, leaving 22,431 chlorpyrifos-exposed and 32,640 nonexposed applicators. Those excluded were older than the total cohort and were more likely to have other missing data. However, they were similar to the rest of the cohort in terms of overall pesticide use.

We used Poisson regression to examine exposure–response relationships within the cohort and to explore the effect of potential confounding factors using the Stata program (version 9.0) (StataCorp. 2005). Relative risks derived from the analysis were adjusted for age at enrollment (< 45, 45–49, 50–54, 55–59, 60–64, 65–69, 70–74, and ≥ 75 years), sex, education (less than high school, high school graduate, greater than high school), smoking (never, former smoker, current smoker), frequency of alcohol drinking during the 12 months before enrollment (no drinking, < 1/month, 1–4/month, > 4/month), and state of residence (Iowa/North Carolina). For category-specific relative risks, we used applicators not exposed to chlorpyrifos as the reference category. Because of potential concomitant exposure to other pesticides, we also adjusted the risk estimates for the four pesticides (alachlor, carbofuran, fonofos, trifluralin) most highly correlated with intensity-weighted exposure-days of chlorpyrifos (Pearson correlation coefficient *r* ≥ 0.4). The exposure levels of these four pesticides were categorized as never, low, and high for regression analysis; for each pesticide, the cut point between low and high exposure was set at the median of intensity-weighted exposure-days among the exposed. We performed trend tests for categorical variables by assigning scores to categories using the median value among cases and treating the scores as continuous variables in the regression analyses. All significance tests were two-sided. We conducted further analyses for a few causes of death stratifying on state of residence, farm type, crop type, farm size, and pesticide application methods to investigate the consistency of associations observed. This analysis used the P1REL0310 release of the Agricultural Health Study data set.

## Results

Table 1 shows selected characteristics of chlorpyrifos-exposed and nonexposed applicators. Among subjects with complete exposure information, 22,431 (41%) reported they had ever used chlorpyrifos. Most of the cohort consisted of male private applicators (mostly farmers), and about 60% of applicators were < 50 years of age. Approximately two-thirds of applicators lived in Iowa, and > 50% were never smokers. Variables in Table 1 showed only small differences between chlorpyrifos-exposed and nonexposed applicators, except for the frequency of use of the four pesticides that were most highly correlated with the use of chlorpyrifos. Chlorpyrifos-exposed applicators also were more likely to live on larger farms (40.5% vs. 33.2%).

A total of 588 and 1,263 deaths were observed among chlorpyrifos-exposed and nonexposed applicators, respectively (Table 2). The overall mortality rate of the chlorpyrifos exposed applicators was slightly less than that of the nonexposed applicators [relative risk (RR) = 0.90; 95% confidence interval (CI), 0.81–1.01]. Although there were no statistically significantly increased risks, any chlorpyrifos exposure was associated with increased risk of death from suicide (ICD-10 codes X60–X84) (RR = 1.45; 95% CI, 0.80–2.63), endocrine, nutritional and metabolic diseases (codes E00–E90) (RR = 1.31; 95% CI, 0.58–2.95), and diseases of the blood and blood-forming organs and certain disorders involving the immune mechanism (codes D50–D89) (RR = 4.40; 95% CI, 0.94–20.45). The risk for death from ischemic heart disease (codes I20–I25) was inversely associated with chlorpyrifos exposure (RR = 0.78; 95% CI, 0.62–0.97) and the risk for lung cancer death (code C34), an *a priori* interest, was not increased (RR = 0.93; 95% CI, 0.64–1.35).

The exposure–response relationships with lifetime exposure days and intensity-weighted exposure-days for chlorpyrifos and seven causes of death are shown in Table 3. The causes of death presented are those with either positive or negative associations in the ever-exposure analyses, or those with some evidence of an exposure–response relation. A positive exposure–response trend was observed for external causes of death (codes V01–Y98) both with lifetime exposure-days and the intensity-weighted exposure days, with a 1.7-fold relative risk in the highest exposure category. Among external causes, number of suicides (codes X60–X84) and non-motor-vehicle accidents (codes W00–X59) increased with lifetime exposure-days, with more than 2-fold relative risks in the highest category. These risks were more increased when we added events such as spills that may have led to unusually high short-term exposures, pesticide poisoning, and depression history to the analysis; however, the results were limited because of the small number of subjects (data not shown). Of 31 non-motor-vehicle accidental deaths, nine were caused by contact with agricultural machinery. This did not account for the association between chlorpyrifos and non-motor-vehicle injury; the relative risk for death due to contact with agricultural machinery (code W30) was 0.87 (95% CI, 0.46–1.66). Of the remaining specific causes, five were caused by falls, four by being struck by objects, two by contact with unspecified machinery, one by drowning, six by accidental poisonings from medications or drugs, and four by external causes likely not related to pesticides, such as fires or other natural disasters. When farm machinery deaths and natural disasters were removed, the risk associated with the remaining accidental deaths was 1.73 (95% CI, 1.04–2.86). Deaths from motor vehicle accidents were not related to pesticide use with either exposure metric (data not shown). Mortality from diseases of the blood and blood-forming organs and certain disorders involving immune mechanisms (codes D50–D89) showed increased risks based on a very small number of exposed cases. No other exposure–response trends were observed among the other causes of death evaluated in our analyses. These results were not changed when we added “total years of pesticide application” to the multivariate analysis as a surrogate measure of other potential farming exposures (data not shown). Results were also similar when we used the low exposure category as the referent group.

To further examine associations with chlorpyrifos, we calculated relative risks for all external causes of death, suicide, and non-motor-vehicle accidents according to lifetime exposure-days stratified by state of residence, farm type, and farm size (Table 4). Non-motor-vehicle accident risks were increased in the highest exposure category in both states, whereas the risks for external cause of mortality and suicide were increased in Iowa only. Risks for suicide tended to be larger for individuals who worked with animals than those who worked with crops; however, these groups were not mutually exclusive. The suicide deaths tended to occur among younger individuals, and the results were similar when the analysis was restricted to deaths before 60 years of age (data not shown). Risk for non-motor-vehicle accidents was greater among those raising crops. Associations between chlorpyrifos use and external causes of death were clearer among applicators from larger farms, although both non-motor-vehicle deaths and suicides were increased for those from small farms in the highest exposure category. The results from analyses stratified by type of crop and pesticide application methods were similar, although our power to detect any differences was limited (data not shown).

## Discussion

We found positive exposure–response trends among pesticide applicators exposed to chlorpyrifos for mortality from external causes (codes V01–Y98), using two exposure measures (lifetime exposure-days and intensity-weighted exposure-days), after controlling for the use of other pesticides and several other potential risk factors in the Agricultural Health Study cohort. Among external cause of death, mortality from suicide (codes X60–X84) and non-motor-vehicle accidents (codes W00–X59) were increased with lifetime exposure-days, with more than 2-fold relative risks in the highest category, although the suicide finding was restricted to Iowa applicators.

Excess suicide mortality has been reported among farmers (Boxer et al. 1995; Gregoire 2002; Lee et al. 2002; London et al. 2005) and workers exposed to pesticides in some studies (Green 1991; van Wijngaarden 2003), but not others (Pickett et al. 1998; Sperati et al. 1999). There was a deficit for suicide among private applicators in the Agricultural Health Study, compared with the general population in Iowa and North Carolina (Blair et al. 2005) after about 5 years of follow-up. This may simply reflect a healthy worker effect or initial self-selection out of the cohort by those who were most seriously depressed. Organophosphate insecticide exposure has been reported to be associated with affective disorders, such as depression (London et al. 2005). Termite applicators exposed to chlorpyrifos reported more neurologic symptoms, including fatigue, loss of muscle strength, and depression (Steenland et al. 2000). A history of pesticide poisoning was associated with depression among spouses in the Agricultural Health Study (Beseler et al. 2006). Animal studies have shown that chlorpyrifos exposure produced permanent alterations in serotonergic mechanisms (Slotkin and Seidler 2005) and changed serotonin-related behaviors (Aldridge et al. 2005) that resemble depression. Previous studies have reported higher rates of depression and anxiety in farmers compared with most other occupational groups (Eisner et al. 1999; Roberts and Lee 1993; Sanne et al. 2004), and depression has consistently been identified as an important risk factor for suicide (Cheng et al. 2000; Inskip et al. 1998).

Although we did not have information on other risk factors for suicide, such as mental illness or financial problems, those factors would not likely differ significantly between chlorpyrifos exposed and nonexposed applicators. Although chlorpyrifos use was more common on large farms, farm size per se was not associated with suicide risk, and the risk of suicide was increased in the highest category of exposure for applicators on small and large farms, although the association appeared stronger on large farms. The risks for suicide among those with chlorpyrifos exposure were more pronounced when we controlled for high pesticide exposure events, pesticide poisoning, and depression history, although we did not have the power to fully explore for these factors.

Farmers are at relatively high risk of work-related injury compared with employees in other industries and occupations in the United States (McCurdy and Carroll 2000; Rautiainen and Reynolds 2002), although mortality from accidents was not excessive compared with the general population in Iowa and North Carolina in the Agricultural Health Study (Blair et al. 2005). Farmers with symptoms of neurotoxicity were reported to be at increased risk of agricultural injury (Atrubin et al. 2005). A recent paper from the Agricultural Health Study cohort suggested that self-reported neurologic symptoms are associated with cumulative exposure to pesticides, including organophosphates (Kamel et al. 2005). Banana workers mildly poisoned with organophosphates, including chlorpyrifos, tended to perform less well on psychomotor and visuomotor tests than did nonpoisoned workers in Costa Rica (Wesseling et al. 2002). Experimental studies also support the possibility that exposure to chlorpyrifos could contribute to acute and long-term injury or accident (Bushnell et al. 2001; Colombo et al. 2005; Moser et al. 2005; Samsam et al. 2005). Previous studies reported that injuries were more frequent on livestock farms than on other types of farm (Browning et al. 1998; Park et al. 2001; Sprince et al. 2003), and among farmers who work on larger farms (Hwang et al. 2001; Layde et al. 1995; Lyman et al. 1999). We observed higher risks for external causes of deaths among applicators from larger farms, but risk for non-motor-vehicle accidents was still increased for applicators from small farms in the highest exposure category. It is possible that farmers on larger farms spend more hours in pesticide mixing and application, resulting in greater pesticide exposure. The risk for all external causes of death was not significantly different between livestock and crop applicators in our study. Furthermore, the association with non-motor-vehicle accidents was stronger for applicators raising crops. The non-motor-vehicle accident association also was not explained by type of equipment used for pesticide application.

We observed an increased exposure–response trend for the ICD category “diseases of the blood and blood-forming organs and certain disorders involving immune mechanisms” (*p* for trend = 0.026 and 0.003, respectively). Among 10 deaths from this cause, nine cases involved specified and unspecified blood disorders including coagulation defects and thrombocytopenia. The small numbers of exposed cases, the non-monotonic shape of the exposure–response curve, and heterogeneity of this death category, however, limit the conclusions that can be drawn.

An association between wheeze and occupational exposure to chlorpyrifos has been reported in the Agricultural Health Study (Hoppin et al. 2002a, 2006). We found no association between mortality from chronic lower respiratory diseases and chlorpyrifos use. Only three deaths were attributed to asthma and all were among nonusers of chlorpyrifos. Death certificates may not accurately reflect asthma prevalence.

We did not see an association between lung or brain cancer mortality and chlorpyrifos exposure, whereas a previous study of cancer incidence in this cohort found significant increased exposure–response trends for lung and brain cancer incidence (Alavanja et al. 2004; Lee et al. 2004). The differences in results may reflect, in part, the use of different exposure cut points. The tertiles used in the prior analysis were based on exposure levels among all chlorpyrifos-exposed applicators with cancer, whereas here the tertiles were based on exposures among all deceased exposed applicators. We carried out additional analyses using the exposure cut points used in the incidence study. However, the small number of deaths in some categories made it difficult to interpret results. The difference may also be attributed to different lag time for data completeness.

Pesticide exposure has been associated with increased risk of Parkinson disease and other neurodegenerative diseases (Baldi et al. 2003a, 2003b; Friedrich 2005; Kamel and Hoppin 2004). However, we did not find any increased risk of death from diseases of the nervous system in chlorpyrifos exposed workers (data not shown). The number of deaths from neurodegenerative diseases was small, and these conditions are often underreported on death certificates.

Unexpectedly, we found a lower risk of ischemic heart disease among applicators ever exposed to chlorpyrifos. Although there is no epidemiologic study focused on chlorpyrifos exposure and circulatory disease, experimental studies have reported that exposure to chlorpyrifos increases blood pressure in rats (Gordon and Padnos 2000; Smith and Gordon 2005). The lack of a monotonic exposure–response pattern and the lack of relevant mechanistic studies weaken the argument for a true protective relation.

A possible limitation of this study is accuracy of the chlorpyrifos exposure histories for those whose use was in the past. Recall of pesticide use by the Agricultural Health Study cohort has been shown to be as reliable as that for other factors routinely evaluated by questionnaire in epidemiologic studies, such as smoking and alcohol use, and to be better than others, such as consumption of fruits and vegetables and physical activity (Blair et al. 2002). In addition, we have found that participants provided plausible information regarding the time period and duration of use of specific pesticides (Hoppin et al. 2002b). A recent report showed that our pesticide exposure algorithm is a reasonably valid measure of exposure intensity compared with urine metabolite monitoring (Coble et al. 2005). Although these findings are encouraging, exposure misclassification undoubtedly occurs. This misclassification would be expected to be nondifferential for cases and noncases in a prospective cohort study and the observed effect estimates would likely be biased toward the null.

Another limitation is that persons who apply pesticides are seldom exposed to just a single agent. Coble et al. (2002) evaluated the relations among different agricultural exposures and found that substantial bias due to unrecognized confounding from exposure to multiple agents was unlikely in this cohort. To reduce the possibility of residual confounding, we adjusted the risk estimates by including in our models the four pesticides most highly correlated with chlorpyrifos. It is not surprising that these pesticides did not confound any association because they are not known to be strong causes of the deaths of interest and they were not tightly linked to chlorpyrifos use, as would be required for strong confounding (Checkoway et al. 2004). There may also be confounding due to other unmeasured differences between those exposed to chlorpyrifos and those who are not exposed. Reassuringly, results were similar when the low exposure category was used as the referent group, and results were unchanged with total years of pesticide use, as a surrogate for other farm-related factors was added to the models.

Another potential weakness is the lack of validity of death certificates for some outcomes. Inaccuracy of death certificates may arise for a variety of reasons, such as incomplete clinical information regarding the circumstances of death or diagnostic errors, and varies by cause of death (Percy et al. 1981). The accuracy of death certificate diagnosis is influenced by the stage of disease at diagnosis and the quality of treatment, which may in turn be influenced by social factors such as education, access to health care, and membership in a health insurance plan. These factors do not apply to causes such as external causes of death. Although death certificates may misclassify some suicides as other external causes of death, death certificates are generally a reliable source of information for external causes of death as a whole. Furthermore, although some outcomes may be underreported, the degree of misclassification is not likely to differ between the exposed and the nonexposed applicators and the bias would therefore be toward the null. We also based our analysis on underlying cause of death. For some outcomes, such as specific respiratory diseases, use of information on contributing causes may provide a more complete picture of patterns of mortality.

Despite these limitations, the Agricultural Health Study has several important strengths. This study is the largest epidemiologic study of applicators exposed to chlorpyrifos conducted to date. All exposure information was collected before the outcome, which reduces concerns regarding bias due to differential reporting among cases and noncases. This study included comprehensive questionnaire data that were used to quantitatively estimate chlorpyrifos exposure levels and to control for potential confounding by lifestyle factors.

In summary, our findings suggest a possible association between chlorpyrifos use and external cause of mortality, such as suicide and non-motor-vehicle accidents, among chlorpyrifos-exposed applicators in the Agricultural Health Study. Although these results might be linked to effects of chlorpyrifos on neurobehavioral function or symptoms, alternate explanations are possible. These findings need to be confirmed in this and other populations, given the limited epidemiologic research on this pesticide.

## Figures and Tables

**Table 1:** Selected characteristics of applicators by chlorpyrifos exposure status based on 1993–1997 enrollment data in the Agricultural Health Study.

Characteristic	Exposed (*n* = 22,431) No. (%)	Nonexposed (*n* = 32,640) No. (%)
Age (years)		
< 45	11,015 (49.1)	14,903 (45.7)
45–49	2,978 (13.3)	3,910 (12.0)
50–54	2,500 (11.1)	3,434 (10.5)
55–59	2,163 (9.6)	3,242 (9.9)
60–64	1,785 (8.0)	2,897 (8.9)
65–69	1,187 (5.3)	2,149 (6.6)
70–74	567 (2.5)	1,332 (4.1)
≥ 75	236 (1.1)	771 (2.3)
Sex		
Male	22,108 (98.6)	31,479 (96.4)
Female	323 (1.4)	1,161 (3.6)
State of residence		
Iowa	15,030 (67.0)	20,928 (64.1)
North Carolina	7,401 (33.0)	11,712 (35.9)
Applicator type		
Private	20,966 (93.5)	29,411 (90.1)
Commercial	1,465 (6.5)	3,299 (9.9)
Smoking history		
Never	11,921 (53.3)	16,922 (52.3)
Former smoker	6,752 (30.2)	9,964 (30.8)
Current smoker	3,675 (16.5)	5,467 (16.9)
Alcohol drinking (drinks per month)		
No	6,494 (29.9)	10,623 (34.2)
< 1	3,463 (16.0)	4,565 (14.7)
1–4	6,744 (31.1)	8,971 (28.9)
> 4	5,003 (23.0)	6,913 (22.2)
Educational level		
Less than high school	1,581 (7.2)	3,575 (11.2)
High school	10,427 (47.5)	15,251 (47.9)
Greater than high school	9,930 (45.3)	12,996 (40.9)
Use of pesticides most highly correlated with use of chlorpyrifos		
Alachlor	13,240 (69.1)	12,309 (38.5)
Carbofuran	7,619 (34.4)	5,100 (15.8)
Fonofos	6,008 (27.0)	4,220 (13.0)
Trifluralin	12,220 (55.3)	13,524 (42.2)
Farm size (acres)		
< 500	12,095 (59.5)	18,570 (66.8)
≥ 500	8,226 (49.5)	9,231 (33.2)

**Table 2:** RRs (95% CIs) for selected causes of death by chlorpyrifos exposure among Agricultural Health Study applicators, 1993–2001.^a^

	No. of deaths		
Cause of death (ICD-10 codes)	Exposed	Nonexposed	RR^b^ (95% CI)
All cause of death	588	1,263	0.90 (0.81–1.01)
All malignant neoplasms (C00–C97)	204	418	0.94 (0.78–1.14)
Colorectal (C18–C20)	26	57	0.95 (0.56–1.61)
Pancreas (C25)	15	25	1.17 (0.58–2.37)
Lung and bronchus (C34)	51	108	0.93 (0.64–1.35)
Prostate (C61)	17	40	0.90 (0.46–1.74)
Brain (C71)	11	12	1.07 (0.41–2.76)
Non-Hodgkin lymphoma (C82–C85)	10	27	0.64 (0.29–1.39)
Leukemia (C91–C95)	12	24	0.89 (0.41–1.92)
Diseases of the blood and blood-forming organs and certain disorders involving the immune mechanism (D50–D89)	5	5	4.40 (0.94–20.45)
Endocrine, nutritional, and metabolic diseases (E00–E90)	12	24	1.31 (0.58–2.95)
Disease of circulatory system (I00–I99)	210	505	0.85 (0.70–1.02)
Ischemic heart disease (I20–I25)	136	341	0.78 (0.62–0.97)
Cardiomyopathy (I42)	13	25	1.06 (0.50–2.23)
Cerebrovascular disease (I60–I69)	20	58	0.84 (0.44–1.62)
Disease of respiratory system (J00–J99)	30	62	0.96 (0.55–1.65)
Chronic lower respiratory disease (J40–J47)	12	39	0.57 (0.25–1.31)
External causes of mortality (V01–Y98)	91	141	1.04 (0.77–1.41)
Motor-vehicle accidents (V01–V99)	33	54	0.93 (0.65–1.32)
Non-motor-vehicle accidents (W00–X59)	32	45	1.04 (0.73–1.48)
Suicide (X60–X84)	24	32	1.45 (0.80–2.63)

a Deaths with < 10 exposed cases are not shown except for diseases of the blood and blood-forming organs and certain disorders involving immune mechanisms (codes D50–D89).

b Relative risks adjusted for age at enrollment, sex, education, smoking, frequency of alcohol drinking during the 12 months before enrollment, and state of residence, and the four pesticides most highly correlated with chlorpyrifos (alachlor, carbofuran, fonofos, trifluralin). The reference category was applicators who were not exposed to chlorpyrifos.

**Table 3:** RRs (95% CIs) for selected causes of death by lifetime chlorpyrifos exposure-days and intensity-weighted chlorpyrifos exposure-days among Agricultural Health Study applicators, 1993–2001.

	All causes of death		External causes of mortality (V01–Y98)		Suicide (X60–X84)		Non-motor-vehicle accidents (W00–X59)		Disease of the blood and blood-forming organs and certain disorders involving immune mechanisms (D50–D89)		Endocrine, nutritional and metabolic diseases (E00–E90)		Ischemic heart disease (I20–I25)	
Chlorpyrifos exposure	No.^a^	RR^b^ (95% CI)	No.	RR (95% CI)	No.	RR (95% CI)	No.	RR (95% CI)	No.	RR (95% CI)	No.	RR (95% CI)	No.	RR (95% CI)
Lifetime chlorpyrifos exposure-days^c^														
Nonexposed	1,263	1.00 (referent )	141	1.00 (referent )	32	1.00 (referent)	45	1.00 (referent)	5	1.00 (referent)	24	1.00 (referent)	341	1.00 (referent)
≤ 20.0	224	0.78 (0.67–0.92)	27	0.74 (0.47–1.17)	9	1.20 (0.53–2.70)	8	0.58 (0.33–1.05)	2	3.59 (0.56–23.22)	3	0.89 (0.26–3.08)	52	0.64 (0.46–0.88)
20.1–56.0	183	0.94 (0.79–1.11)	28	0.90 (0.57–1.42)	5	1.01 (0.38–2.70)	9	0.84 (0.48–1.46)	1	2.76 (0.25–30.41)	5	1.75 (0.57–5.39)	53	0.99 (0.72–1.38)
≥ 56.1	158	1.10 (0.91–1.33)	32	1.69 (1.10–2.59)	9	2.37 (1.03–5.48)	14	2.20 (1.36–3.52)	2	10.11 (1.48–68.95)	3	1.82 (0.51–6.54)	29	0.83 (0.54–1.26)
*p*_trend_		0.205		0.008		0.044		0.036		0.026		0.329		0.520
Intensity-weighted chlorpyrifos exposure-days^d^														
Nonexposed	1,263	1.00 (referent)	141	1.00 (referent)	32	1.00 (referent)	45	1.00 (referent)	5	1.00 (referent)	24	1.00 (referent)	341	1.00 (referent)
≤ 78.8	167	0.84 (0.70–1.00)	18	0.59 (0.33–1.04)	7	1.18 (0.47–2.98)	5	0.42 (0.20–0.89)	0	—	3	1.30 (0.37–4.56)	36	0.64 (0.44–0.93)
78.9–318.1	159	0.88 (0.74–1.06)	24	0.91 (0.56–1.47)	4	0.87 (0.30–2.58)	8	0.93 (0.53–1.63)	0	—	2	1.07 (0.24–4.75)	44	0.92 (0.65–1.30)
≥ 318.2	168	1.00 (0.82–1.20)	37	1.71 (1.13–2.60)	9	2.05 (0.86–4.84)	16	2.09 (1.30–3.36)	3	12.68 (1.88–85.42)	5	2.42 (0.75–7.82)	33	0.78 (0.52–1.19)
*p*_trend_		0.005		0.005		0.101		0.034		0.003		0.146		0.316

a Number of deaths; the reduced number of subjects included in the intensity-weighted exposure-days analysis is attributed to occasional missing data for some components of the intensity algorithm.

b Relative risks adjusted for age at enrollment, sex, education, smoking, frequency of alcohol drinking during the 12 months before enrollment, and state of residence, and the four pesticides most highly correlated with chlorpyrifos (alachlor, carbofuran, fonofos, trifluralin). The reference category was applicators who were not exposed to chlorpyrifos. Applicators who did not provide information on chlorpyrifos days per year, years of use, and intensity level were excluded from this analysis.

c Lifetime exposure-days = years of use × days per year. Cut points based on the distribution of all deaths among chlorpyrifos-exposed applicators.

d Intensity-weighted exposure-days = years of use × days per year × intensity index. Cut points based on the distribution of all deaths among chlorpyrifos-exposed applicators.

**Table 4:** RRs (95% CIs) for external causes of mortality by lifetime chlorpyrifos exposure-days among Agricultural Health Study applicators stratified by state of residence and farm type, 1993–2001.

	External causes of mortality (V01–Y98)		Suicide (X60–X84)		Non-motor-vehicle accidents (W00–X59)	
Stratification variable, chlorpyrifos exposure	No. of deaths^a^	RR^b^ (95% CI)	No. of deaths	RR (95% CI)	No. of deaths	RR (95% CI)
State of residence						
Iowa						
Nonexposed	68	1.0 (referent)	12	1.0 (referent)	25	1.0 (referent)
≤ 20.0	17	0.93 (0.53–1.65)	4	1.41 (0.43–4.57)	8	0.88 (0.48–1.63)
20.1–56.0	18	1.15 (0.66–2.00)	3	1.21 (0.33–4.47)	7	1.08 (0.58–2.00)
≥ 56.1	22	2.36 (1.41–3.95)	6	3.93 (1.41–10.96)	8	1.95 (1.06–3.58)
*p*_trend_		0.001		0.008		0.023
North Carolina						
Nonexposed	73	1.0 (referent)	20	1.0 (referent)	20	1.0 (referent)
≤ 20.0	10	0.53 (0.24–1.17)	5	1.04 (0.34–3.21)	0	—
20.1–56.0	10	0.56 (0.23–1.33)	2	0.85 (0.19–3.87)	2	0.31 (0.07–1.34)
≥ 56.1	10	0.93 (0.42–2.02)	3	0.93 (0.19–4.57)	6	2.14 (1.00–4.60)
*p*_trend_		0.966		0.921		0.007
Farm type^c^						
Crop						
Nonexposed	56	1.0 (referent)	15	1.0 (referent)	11	1.0 (referent)
≤ 20.0	9	0.60 (0.25–1.43)	2	0.43 (0.05–3.34)	3	1.28 (0.49–3.34)
20.1–56.0	9	0.87 (0.38–1.98)	1	0.63 (0.08–5.03)	2	0.50 (0.11–2.19)
≥ 56.1	12	1.57 (0.78–3.24)	4	1.95 (0.50–7.58)	7	4.36 (2.00–9.53)
*p*_trend_		0.155		0.268		0.001
Animal						
Nonexposed	75	1.0 (referent)	14	1.0 (referent)	29	1.0 (referent)
≤ 20.0	15	0.70 (0.39–1.26)	6	1.62 (0.58–4.52)	4	0.34 (0.14–0.80)
20.1–56.0	17	0.82 (0.45–1.49)	3	1.10 (0.30–4.06)	7	0.94 (0.51–1.74)
≥ 56.1	20	1.79 (1.05–3.06)	5	2.96 (0.96–9.07)	7	1.49 (0.80–2.81)
*p*_trend_		0.016		0.072		0.095
Farm size (acres)						
< 500						
Nonexposed	89	1.0 (referent)	18	1.0 (referent)	31	1.0 (referent)
≤ 20.0	16	0.56 (0.30–1.04)	5	1.02 (0.33–3.17)	4	0.35 (0.15–0.82)
20.1–56.0	15	0.68 (0.34–1.33)	2	0.85 (0.19–3.83)	4	0.58 (0.25–1.37)
≥ 56.1	13	1.32 (0.70–2.50)	3	1.52 (0.33–6.97)	6	1.99 (1.03–3.83)
*p*_trend_		0.768		0.786		0.640
≥ 500						
Nonexposed	27	1.0 (referent)	6	1.0 (referent)	8	1.0 (referent)
≤ 20.0	9	1.25 (0.56–2.78)	3	1.54 (0.36–6.58)	4	1.43 (0.58–3.51)
20.1–56.0	10	1.30 (0.60–2.82)	2	1.06 (0.21–5.42)	4	1.17 (0.47–2.90)
≥ 56.1	18	3.00 (1.52–5.92)	5	3.30 (0.92–11.78)	8	3.14 (1.43–6.89)
*p*_trend_		0.004		0.121		0.012

a The reduced number of subjects included in the intensity-weighted exposure-days analysis is attributed to occasional missing data for some components of the intensity algorithm.

b Relative risks adjusted for age at enrollment, sex, education, smoking, frequency of alcohol drinking during the 12 months before enrollment, and state of residence, and the four pesticides most highly correlated with chlorpyrifos (alachlor, carbofuran, fonofos, trifluralin). The reference category was applicators who were not exposed to chlorpyrifos. Applicators who did not provide information on chlorpyrifos days used per year, years of use, and intensity level were excluded from this analysis.

c The variable was generated using the information on beef cattle, dairy cattle, hogs/swine, poultry, sheep, eggs, other farm animals, and type of crop in the enrollment questionnaire. The categories are not mutually exclusive.
